# Validation of a mouse xenograft model system for gene expression analysis of human acute lymphoblastic leukaemia

**DOI:** 10.1186/1471-2164-11-256

**Published:** 2010-04-21

**Authors:** Amy L Samuels, Violet K Peeva, Rachael A Papa, Marin J Firth, Richard W Francis, Alex H Beesley, Richard B Lock, Ursula R Kees

**Affiliations:** 1 Division of Children's Leukaemia and Cancer Research, Telethon Institute for Child Health Research, Perth, Western Australia; 2 Centre for Child Health Research, The University of Western Australia, Perth, Western Australia; 3 Leukaemia Biology, Children's Cancer Institute Australia for Medical Research, Randwick, New South Wales, Australia; 4 Division of Bioinformatics and Biostatistics, Telethon Institute for Child Health Research, Perth, Western Australia

## Abstract

**Background:**

Pre-clinical models that effectively recapitulate human disease are critical for expanding our knowledge of cancer biology and drug resistance mechanisms. For haematological malignancies, the non-obese diabetic/severe combined immunodeficient (NOD/SCID) mouse is one of the most successful models to study paediatric acute lymphoblastic leukaemia (ALL). However, for this model to be effective for studying engraftment and therapy responses at the whole genome level, careful molecular characterisation is essential.

**Results:**

Here, we sought to validate species-specific gene expression profiling in the high engraftment continuous ALL NOD/SCID xenograft. Using the human Affymetrix whole transcript platform we analysed transcriptional profiles from engrafted tissues without prior cell separation of mouse cells and found it to return highly reproducible profiles in xenografts from individual mice. The model was further tested with experimental mixtures of human and mouse cells, demonstrating that the presence of mouse cells does not significantly skew expression profiles when xenografts contain 90% or more human cells. In addition, we present a novel *in silico *and experimental masking approach to identify probes and transcript clusters susceptible to cross-species hybridisation.

**Conclusions:**

We demonstrate species-specific transcriptional profiles can be obtained from xenografts when high levels of engraftment are achieved or with the application of transcript cluster masks. Importantly, this masking approach can be applied and adapted to other xenograft models where human tissue infiltration is lower. This model provides a powerful platform for identifying genes and pathways associated with ALL disease progression and response to therapy *in vivo*.

## Background

Understanding the complex molecular pathways leading to disease is critical for the development of effective treatment regimes and novel drug targets. Due to research and resource limitations associated with the use of primary patient material, pre-clinical models are essential to expand our knowledge of cancer biology and for the evaluation of new drugs. For pre-clinical testing, cell lines cultured *in vitro *have been extensively used but their ability to recapitulate primary disease is limited. Therefore, more relevant disease models are of critical importance.

An ideal model would mimic the proliferation and dissemination of cancer cells that occur *in vivo *and behave in a similar manner in response to chemotherapeutic drug treatment. The non-obese diabetic/severe combined immunodeficient (NOD/SCID) xenograft mouse model is currently one of the most successful models with which to study haematological malignancies such as acute lymphoblastic leukaemia (ALL) [1], whereby patient bone marrow leukaemia cells are directly transplanted into recipient NOD/SCID mice [2]. The kinetics of engraftment reflects the human disease, leading to bone marrow (BM) infiltration, followed by migration to the spleen, peripheral blood and other haematopoietic organs [2-4].

For ALL, although cure rates are exceeding 75%, the development of drug resistance is poorly understood and remains a major cause of morbidity and mortality in children [5]. Importantly, much of our knowledge of the mechanisms underlying drug resistance has been generated *in vitro *using immortalised cancer cell lines. The extent to which cell lines retain features of the original disease *in vivo *is a matter of debate [6]. Thus relevant *in vivo*, pre-clinical models that recapitulate human disease are critical to delineate resistance mechanisms and improve survival.

Primary leukaemia cells engrafted into NOD/SCID mice appear to retain many of the phenotypic and genotypic features of the original specimen [2,7-10]. Moreover, their drug resistance profile to conventional chemotherapeutics mirrors that of the patient clinical response [2,10]. Importantly, comparisons have shown that such xenografts more closely resemble their tumour type of origin than *in vitro *cell lines and have been accurate in predicting efficacious drug combinations and clinically active therapeutics [11-14].

Continuous xenografts can be established by transplanting cells harvested from the spleen of engrafted animals into secondary and tertiary recipient mice [10]. Utilising continuous ALL NOD/SCID xenografts the effects of chemotherapy drugs can be assessed at the molecular level. Thus, the aim of the current study was to characterise gene expression profiling in the continuous ALL xenograft so that it can be used as a model for the development of therapy resistance *in vivo*. We have previously demonstrated the clinical relevance of gene-expression profiling through the successful identification of markers predictive of ALL disease outcome, drug-resistance and relapse in a number of primary ALL patient cohorts [15-21]. However, to validate the xenograft model system for transcriptional analysis three critical issues needed to be addressed. Firstly, we needed to determine the most appropriate engrafted xenograft tissue for analysis. BM is more commonly isolated from patients, however, the spleen in xenograft mice contains at least seven-fold more leukaemia cells, which makes isolation of these cells more practical for analysis. Thus, we were interested in establishing whether the same gene expression profiles can be obtained from engrafted spleen and BM. Secondly, we wanted to address the reproducibility of the engraftment in both the BM and spleen of independent mice. Phenotypic evidence suggests engraftment in the continuous mouse model is reproducible [10], however, this has not been examined at the transcriptional level. Finally, when testing the expression of human xenografts we wanted to measure the effect of the host murine tissue. Studies from other xenograft models have demonstrated cross-species hybridisation of mouse RNA to human specific microarrays. Although not extensively characterised, such studies suggest the potential skewing of human gene expression profiles [22-26]. The previous studies were performed using Affymetrix expression arrays designed to target the 3' end of the gene. This region shows the most divergence between mouse and human. To date, the extent of cross hybridisation using the Affymetrix whole transcript Human Gene 1.0 ST array has not been assessed. The goal of the present study was therefore to validate the use of gene expression profiling in the ALL NOD/SCID xenograft model so that it can be used as a pre-clinical model of relapse.

## Results

### Comparison of BM/spleen transcriptional profiles of ALL xenografts

**Figure 1:**
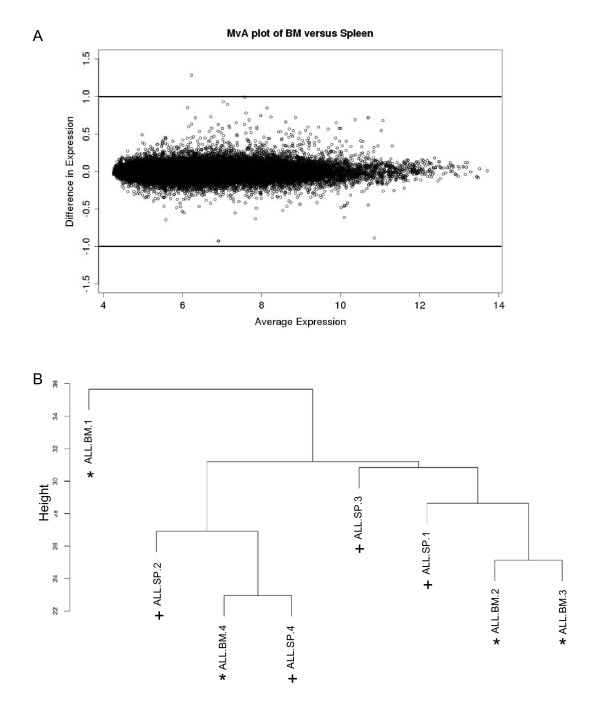
**Comparison of differential expression levels between engrafted ALL-16 bone marrow (n = 4) and spleen (n = 4) xenografts**. **A **MvA plot comparing expression differences with expression averages for all transcript clusters. Plots were generated using RMA normalised log_2 _signal intensities, lines represent 2-fold difference (log_2 _= 1). **B **Unsupervised hierarchical clustering of engrafted ALL-16 bone marrow (*) and spleen (**+**) xenografts from four NOD/SCID mice.

Our previous studies have demonstrated that there are no morphological differences in ALL cells engrafted to the BM or spleen of NOD/SCID mouse xenografts [2]. We therefore conducted a microarray investigation to determine if the transcriptional profiles for engrafted spleen and BM are also analogous. The previously described continuous ALL-16 xenograft established in NOD/SCID mice was used to obtain engrafted BM and spleen tissue [2]. At harvest, high levels of human CD45+ cell engraftment were reached with > 99% human cells infiltrating the mouse spleen and BM. Using Affymetrix GeneChip Human Gene 1.0 ST^® ^arrays the transcriptional profile of engrafted BM tissue was compared to spleen tissue following normalisation. We compared BM and spleen from four engrafted mice. Pearson correlation coefficients were calculated and MvA plots, which display the log difference between BM and spleen expression levels on the y-axis to the average signal intensity on the x-axis, were generated. The MvA plot demonstrates little variation in expression levels between the BM and spleen arrays, only one transcript cluster showed a two fold difference in expression (Figure 1A). The Pearson correlation coefficients calculated between BM and spleen further show high concordance, ranging between 0.99234-0.99683 for the four xenografts (Table 1). Consistent with these results unsupervised hierarchical clustering revealed that the xenografts do not cluster according to BM or spleen tissue origin (Figure 1B). To determine differential expression between engrafted BM and spleen a moderated t-test was performed. To account for the small sample size a linear model with empirical Bayes estimation (limma) and Benjamini-Hochberg correction was applied [27,28]. With this approach only three of the 28,869 genes profiled, *ATP binding cassette A *(*ABCA1*), *ATP binding cassette G *(*ABCG1*) and *insulin-like growth factor binding protein 5 *(*IGFBP5*) were identified as significantly differentially expressed (p < 0.05). Together these results demonstrate a high level of transcriptional concordance between engrafted BM and spleen suggesting both could be used interchangeably for gene expression analysis.

**Table 1:** Pearson correlation summary statistics showing the engraftment reproducibility between bone marrow and spleen xenografts from four independent mice.

	**ALL.BM - ALL.BM**	**ALL.SP -ALL.SP**	**ALL.BM - ALL.SP**
Range	0.99394	0.99445	0.99234
	0.99657	0.99576	0.99683
n (comparisons)	6	6	16
SD	0.00102	0.00052	0.00120
Mean	0.99556	0.99529	0.99496
SE	0.00041	0.00021	0.00030

ALL = Acute lymphoblastic leukaemia, BM = bone marrow, SP = spleen, SE = standard error.

### Reproducibility of engraftment

To establish the reproducibility of engraftment in the continuous ALL model, we compared the gene expression profiles of ALL-16 xenografts from the spleen and BM of four independent mice. Pearson correlation coefficients were calculated to compare the reproducibility of the mean signal intensity between biological replicates (Additional file 1). All transcript clusters were compared for pairwise combinations of microarrays (Table 1) and correlations between tissues showed little variation. These results demonstrate excellent reproducibility in the transcriptional profiles of independent mice transplanted with the ALL-16 xenograft. Importantly, this high level of reproducibility was observed when comparing BM or spleen xenografts among independent mice. This high concordance suggests engraftment of leukaemia cells in BM and spleen is highly reproducible in the continuous ALL xenograft model.

### Analysis of cross species hybridisation in the ALL xenograft model

Recovery of human tissue from the engrafted murine host introduces the possibility of contamination from the host mouse cells. The ALL xenograft mouse represents a high engraftment model. At harvest, BM, spleen and peripheral blood all reach high levels of human CD45+ cells, with BM and spleen typically containing > 97% (with a range from 90-99%) [2]. Therefore, BM/spleen xenograft preparations are estimated to contain 1-10% mouse cells. We developed an experimental approach to evaluate the effect of residual mouse cells in the presence of 90-100% human ALL cells using the whole transcript Affymetrix microarray platform.

**Figure 2:**
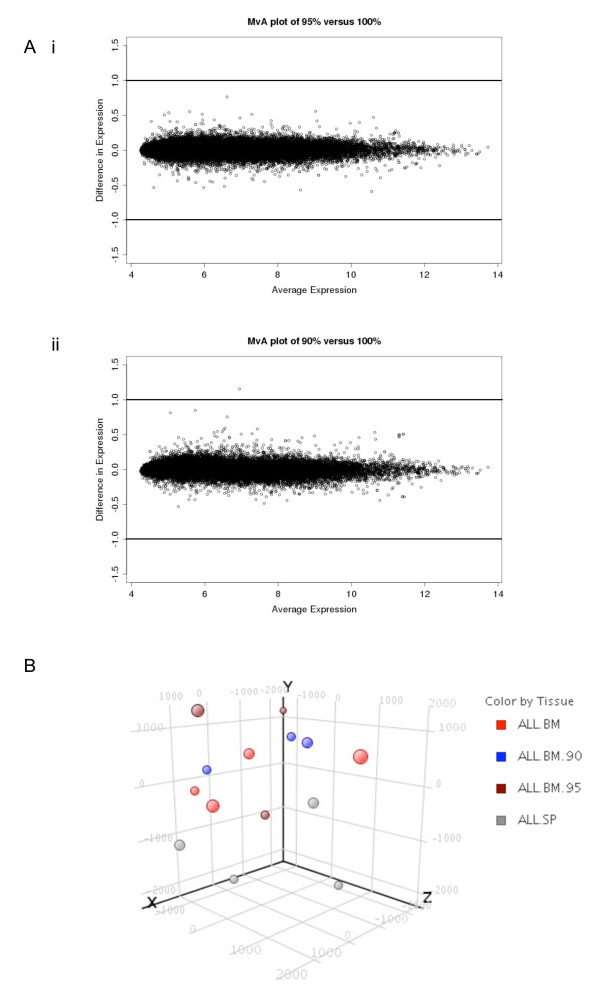
**Assessment of differential expression after mixing ALL-16 xenografts with mouse cells (NES)**. **A **MvA plots comparing **i **ALL.100 (100% human ALL) (n = 8) with ALL.95 (95% human ALL) (n = 3) and **ii **ALL.100 with ALL.90 (90% human) (n = 6), MvA plot comparing expression differences with expression averages for all transcript clusters. Plots were generated using RMA normalised log_2 _signal intensities, lines represent 2-fold difference (log_2 _= 1). **B **Principle component analysis of global gene-expression profiles from ALL engrafted bone marrow, spleen tissues and cell mixtures of ALL.95 (95% human) and ALL.90 (90% human). Principle components were extracted using all transcript clusters. Four principle components were calculated, of which the first three are shown.

Graded cell mixtures were prepared with 100% engrafted human leukaemia cells (ALL.100) and spleen cells prepared from non-engrafted mice (NES) to derive 95% human (ALL.95) and 90% human (ALL.90) cell mixtures. All three mixtures were analysed by microarray in triplicate to detect transcriptional changes due to mouse RNA. When comparing ALL.100, with ALL.95 or ALL.90 highly concordant gene expression profiles were obtained with little variation shown by MvA plots of the mean signal intensity (Figure 2A). Moreover, Pearson correlation coefficients comparing ALL.100 with ALL.95 or ALL.90 showed no difference between the cell mixtures (Table 2). Principal components analysis (PCA) demonstrated that there is no clustering between any of the samples including the three biological replicates for each cell mixture, further showing the high degree of similarity between all samples examined (Figure 2B). To compare differential gene expression a moderated t-test (limma) with Benjamini-Hochberg correction was performed. This approach did not identify any significant (p < 0.05) differential gene expression between ALL.100 and ALL.95 or ALL.90. These results indicate that the presence of <10% mouse cells does not significantly skew human gene expression signatures. Importantly, other xenograft models show different levels of mouse cells in engrafted tissue and therefore may require a masking approach to control cross-species hybridisation. We tested this using two complementary masking approaches.

**Table 2:** Pearson correlation summary statistics showing the reproducibility of mixing experiments between ALL.90 (90% human ALL) (n = 3) versus ALL.100 (100% human ALL) (n = 8) and ALL.95 (95% human ALL) (n = 3) versus ALL.100 from independent mice.

	**ALL.90 - ALL.100**	**ALL.95 - ALL.100**
Range	0.99316	0.99247
	0.99731	0.99712
n (comparisons)	24	24
Mean	0.99537	0.99527
SE	0.00021	0.00028

ALL = Acute lymphoblastic leukaemia, SE = standard error.

### Masking cross species hybridisation

**Figure 3:**
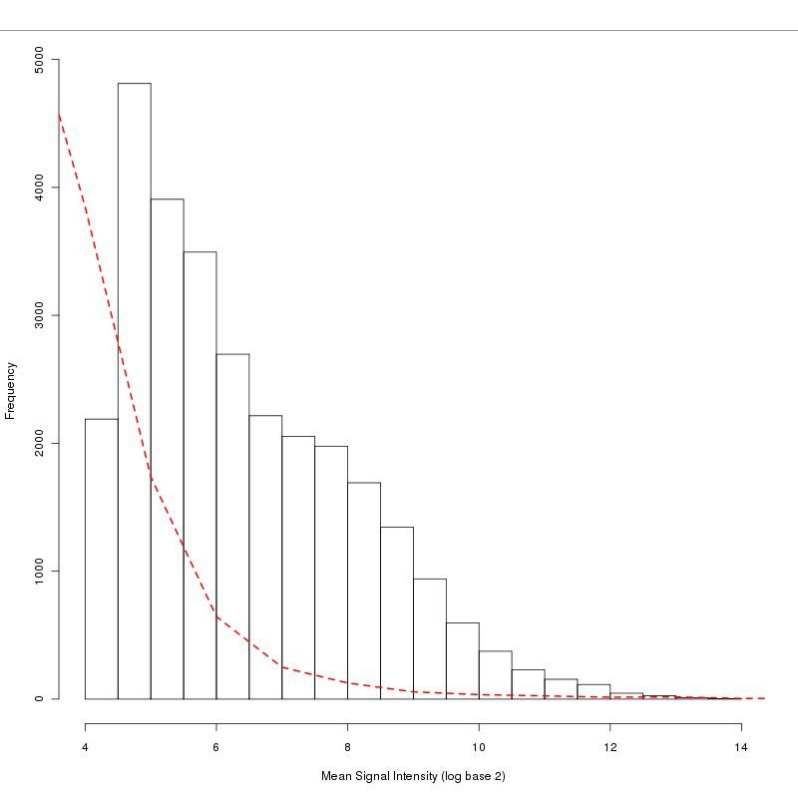
**Histogram comparing mean signal intensity of engrafted ALL-16 xenografts and mouse non engrafted spleen (NES)**. Frequency and RMA normalised log_2 _mean signal intensity for all transcript clusters. The histogram compares ALL.100 (100% human ALL) (n = 8) represented by bars and mouse NES (n = 3) represented by a dashed line.

**Figure 4:**
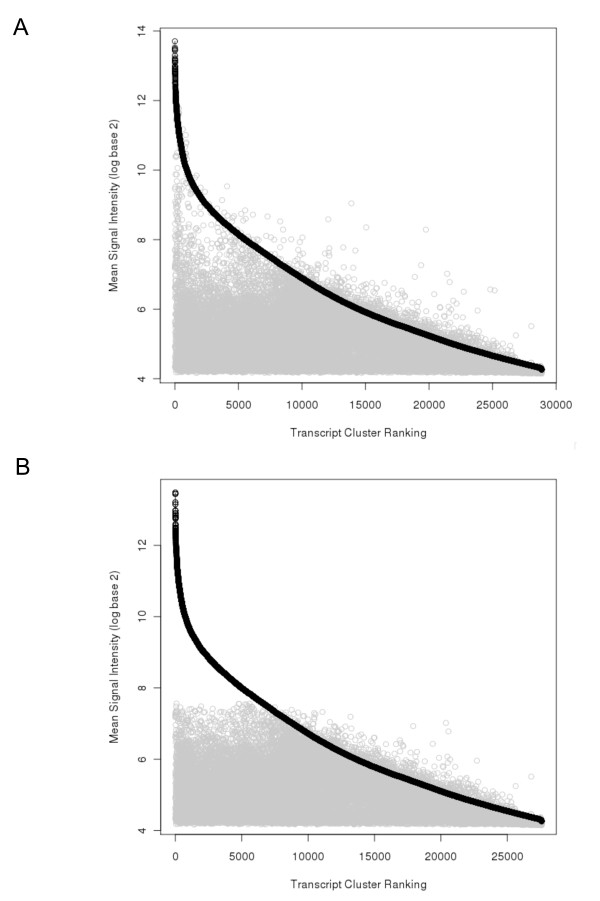
**Comparison of ALL.100 ranked transcript cluster signal intensity to mouse non engrafted spleen (NES)**. Comparison of ranked ALL.100 (100% human ALL) (n = 8), shown in black, and corresponding mouse NES (n = 3), shown in grey, mean signal intensity. Transcript clusters are ranked according to descending mean signal intensity of ALL.100. **A **Without application of filter **B **Filtered, via the removal of mouse NES transcript clusters from the top 1% of normalised expression with a mean signal intensity > 7.56 (log base 2 scale).

We first tested the hybridisation of pure mouse non-engrafted spleen (NES) to human arrays (Figure 3). We found that the overall raw signal intensity and signal to noise ratio (SNR) of mouse transcripts was significantly reduced compared to the hybridisation of the human samples (signal intensity 115.36-329.47 and SNR 0.7-1.67 respectively). The signal distribution of the hybridising targets showed a reduced frequency for high intensity values for mouse NES compared to ALL.100 (Figure 3). A small percentage of transcript clusters, however, resulted in very high signals for the mouse NES samples, these were selected as candidates for the masking approach (Figure 4). Taking the top 1% of normalised mean signal expression we identified 312 transcript clusters with a signal intensity higher than 7.56 (log base 2 scale) (Figure 4B) (Additional file 2).

We then identified transcript clusters theoretically susceptible to cross-species hybridisation due to their associated probes having high identity to mouse transcripts. We identified 75,300 probes, which matched the mouse genome with 100% identity and/or with a single base mismatch. Of the 28,869 human transcript clusters targeted by the Human Gene ST 1.0 array our filtering method revealed 1,085 (~3.75%) transcript clusters as candidates susceptible to cross-species hybridisation (Additional file 2).

**Figure 5:**
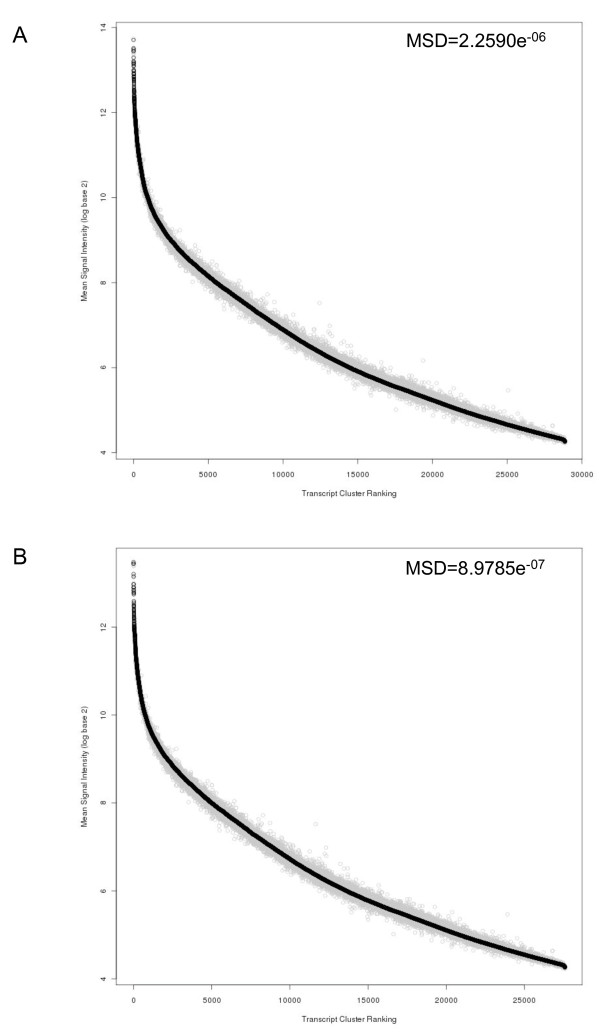
**Comparison of ALL.100 ranked transcript cluster signal intensity to ALL.90**. Comparison of ranked ALL.100 (100% human ALL) (n = 8) shown in black and corresponding ALL.90 (90% human) (n = 3), shown in grey, mean signal intensity. Transcript clusters are ranked according to descending mean signal intensity of ALL.100. **A **Without experimental mask **B **With application of experimental mask. Mean square distance (MSD) was measured between each ALL.100 transcript cluster and the corresponding ALL.90 transcript cluster.

**Figure 6:**
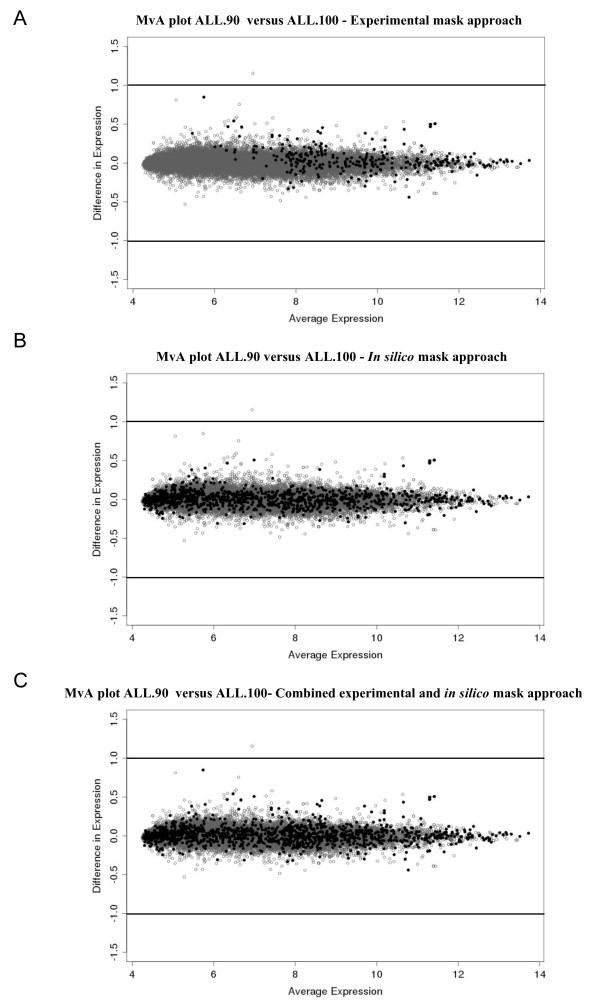
**Application of *in silico *and experimental masks**. MvA plots comparing expression differences with expression averages for all transcript clusters. Expression signals from ALL-16 xenografts ALL.100 (100% human) (n = 8) versus ALL.90 (90% human) (n = 3) were compared following the application of the **A **experimental **B ***in silico *or **C **combined masking approaches. Transcript clusters identified as susceptible to cross-species hybridisation are shown in black (.) all others are grey (.). Plots were generated using RMA normalised log_2 _signal intensities, lines represent 2-fold difference (log_2 _= 1).

Of the 312 experimentally derived transcript clusters, 117 (37.5%) were also identified using the *in silico *approach, highlighting a degree of overlap between the two approaches. Both the experimental and *in silico *masking approaches were combined resulting in 1280 (4.4%) transcript clusters to be masked from the total gene expression signals (Additional file 2). The combined masking approach was applied to ALL.90 and compared to the ranked signal intensity of ALL.100 (Figure 5). Following application of the combined mask, calculation of the mean squared distance (MSD) between ALL.100 and ALL.90 revealed a decrease in variability (MSD = 2.259019e^-06^-8.978447e^-07 ^respectively). Using MvA plots we applied each of the *in silico*, experimental and combined masking approaches to the ALL.100 versus ALL.90 comparison (Figure 6). Application of the combined approach removed several of the outliers and thus variability enabling us to reduce cross-species hybridisation and improve concordance between the ALL.100 and ALL.90 transcriptional profiles.

## Discussion

The current study sought to address the sensitivity of gene expression profiling for human xenografts growing in mice without prior cell separation. Mouse cells, present in xenografts can be separated using lengthy depletion protocols and fluorescent activated cell sorting (FACS) which can have deleterious affects on transcriptional profiles. Several studies, using a variety of xenograft mouse models, have previously examined cross-species hybridisation without prior cell separation [22-26]. These studies were performed using Affymetrix expression arrays designed to target the 3' end of genes. The 3' expression arrays are thought to be relatively insensitive to cross-species hybridisation as there is greater than 10% DNA divergence between mouse and human within the 3' untranslated region (UTR). In contrast, the Human gene 1.0 ST array queries the whole transcript, giving a more accurate reflection of gene expression. Using the whole transcript platform, our results show that mouse cells do not significantly skew cancer expression profiles when xenografts contain 90% or more human cells. Although mouse RNA was found to cross-hybridise to the whole transcript arrays in the absence of human target, when tested in the presence of human RNA (> 90%), the effect on the transcriptional profiles was minimal. Our microarray analysis and theoretical modelling indicated that the design of the whole transcript Affymetrix chips is effective enough to limit aberrant cross-hybridising mouse RNA. Therefore, without prior cell separation, profiling xenografts on whole transcript arrays is possible for high engraftment haematological models.

We developed experimental and *in silico *derived gene sets to mask probes and transcript clusters potentially susceptible to cross-species hybridisation. Application of the transcript cluster masks to the gene expression profiles of the human/mouse graded mixtures, improved results by removing outliers and reducing variability. While the derived masking approaches outlined in this study were used for a high engraftment model, this masking approach could be applied and adapted to other xenograft model systems where human tissue infiltration is below 90%.

The kinetics of engraftment in the NOD/SCID ALL xenograft model reflect the human disease with BM infiltration, followed by migration to the spleen, peripheral blood and other haematopoietic organs [2-4]. Importantly, our findings suggest that spleen is a practical alternative to BM for profiling xenograft tissue. Microarray analysis revealed no significant difference in the transcriptional signatures of engrafted BM and spleen. Moreover, we determined that the process of leukaemia engraftment is highly concordant and reproducible between independent mice.

## Conclusions

Characterisation of reliable pre-clinical models to identify the most promising new agents to enter clinical trials and molecular events underlying drug resistance is of critical importance. For childhood cancers, xenograft models have been particularly accurate in identifying clinically active agents and effective drug combinations. The continuous NOD/SCID xenograft model for ALL provides a powerful platform with which to study and monitor drug resistance phenotypes *in vivo*. In this report we have evaluated the accuracy of molecular signatures derived from xenografts. We demonstrate that species-specific transcriptional profiles can be obtained from xenografts when high levels of engraftment are achieved or with the application of transcript cluster masks. Ultimately, using the continuous xenograft model, this experimental approach will allow the molecular analysis of the effects of individual drugs and drug combinations as well as the identification of biological mechanisms associated with drug resistance phenotypes *in vivo*.

## Methods

### T-ALL Xenograft mouse model

The University of New South Wales Animal Care and Ethics Committee approved all experimental procedures involving NOD/SCID mice. The ALL-16 xenograft line was established from a patient T-ALL diagnosis sample in the Non-Obese Diabetic/Severe Combined Immunodeficient (NOD/SCID) mouse line as previously reported [2]. Briefly, cells harvested from the spleens of engrafted animals were transplanted into secondary, tertiary and quaternary recipient mice to establish a continuous xenograft model. Quaternary xenografts were used in this study. Engraftment and disease progression was monitored by flow cytometric enumeration of the proportion of human versus mouse CD45+ (%huCD45+) cells in peripheral blood using established procedures [2,10]. When the %huCD45+ reached 50%, animals were culled and tissue was harvested.

### Sample Preparation

Tissue harvested was femoral bone marrow and spleen from xenografted animals, and spleen from non-engrafted mice. Mononuclear cells from spleen samples were purified by syringe homogenisation, followed by filtration and density gradient centrifugation by Ficoll-Paque (n = 4) (LymphoPrep, Nycomed). Bone marrow cells were collected by flushing both femurs with RPMI 1640 medium (Life Technologies) into a collection tube without density gradient centrifugation (n = 4). All cells were cryopreserved for future use in FCS containing 10% DMSO.

Recovery of human tissue from the engrafted mouse introduces the possibility of contamination from mouse cells. To investigate this further, 95% and 90% cell mixtures were prepared using T-ALL from engrafted femoral bone marrow with the non-engrafted mouse spleen from the NOD/SCID mouse strain (n = 3).

### RNA Isolation

Total RNA was extracted using TRIZOL reagent (Invitrogen) followed by purification with the RNeasy Mini kit (Qiagen). Samples were ethanol precipitated and quantitated by Nanodrop ND-1000 Spectrophotometry (Thermo Scientific). RNA integrity was assessed using the Agilent 2100 Bioanalyzer and the RNA Nano 6000 kit (Agilent Technologies).

### Labelling and Hybridisation

Samples were labelled according to manufacturers instructions for the GeneChip WT Sense Target Labelling Protocol (Affymetrix). Briefly, 300 ng of total RNA was reverse transcribed into double stranded cDNA using T7-(N)_6 _random primers. An *in vitro *transcription step was carried out overnight to generate cRNA. Using the cRNA as a template we generated cDNA, which was then fragmented, labelled and hybridised onto Affymetrix Genechip Human Gene 1.0 ST Arrays. The arrays interrogate 28 869 well-annotated genes with 764 885 probes. The design of the Human Gene 1.0 ST Array was based on the UCSC hg18 and NCBI Build 36 genome sequence assembly. Arrays were washed and stained using the GeneChip Fluidics Station 450 and scanned with the GeneChip Scanner 3000 7G (Affymetrix). All arrays passed quality control criteria as assessed by Expression Console software (Affymetrix).

### Data Analysis

Raw data CEL files were normalised using the Robust Multi-Array (RMA-16) algorithm in GeneSpring version GX 10.0.2 (Agilent Technologies). A mean adjustment of the normalised signal intensity was carried out to account for minor batch effects seen in the data. Analysis was performed using R version 2.6.2 [29,30]. Microarray data from this study can accessed from ArrayExpress, accession number E-MEXP-2648 http://www.ebi.ac.uk/arrayexpress

To enable statistically significant data to be interpolated from small sample sizes we used the limma package from Bioconductor [27]. The data was fitted to a linear model and the probability of differential expression was estimated using an empirical Bayes moderated t-test. The p-values were adjusted for multiple testing using a Benjamini-Hochberg correction [28]. Unsupervised hierarchical clustering and Pearson Correlations were generated using the log_2 _signal intensities following normalisation. MvA plots were generated in R using the average over the replicates in each group. Mean squared distance (MSD) were calculated in R, ranked transcript cluster signal intensity plots and mean signal histograms were also generated in R. Principal Components Analysis (PCA) based on samples and four components was generated in GeneSpring.

### Development of a cross-species making approach

To filter cross-species hybridisation we identified all oligonucleotide probes on the Human GeneChip 1.0 ST array susceptible to spurious signal from mouse RNA using a coupled bioinformatics, *in silico *and experimental masking approach which is detailed below.

### Cross-species hybridisation derived *in silico*

A murine genomic sequence database was compiled from release 54 of the Ensembl Database [31]. All HuGene array annotation files were obtained directly from the Affymetrix website [32]. The probes, probesets and transcript clusters from these files annotated as targeting core human transcripts (i.e. non Affymetrix control probes) were combined, based on their annotated genomic coordinates, to create a mapping table with a three tier hierarchy where probes belong to probesets that in turn belong to transcript clusters (mapping table available on request). The nucleotide sequences for each probe on the HuGene array were obtained from the Affymetrix probe annotation file. The alignment tool BLAT was used to map human probe sequences to the murine genome sequence database. Probes were considered a hit if they matched a database sequence with 100% identity or with a single base mismatch. The BLAT parameters were optimised to detect the 25 mer probes [33] with tileSize and stepSize set to 12 and 7 respectively. An in house designed Perl script was then used in conjunction with our mapping table to parse the BLAT output files in order to filter out those transcript clusters containing probesets with large numbers of hit probes. Transcript clusters with high numbers of associated hit probes would be deemed candidates for masking. In this filtering process, probes were systematically removed from any probesets with which they were associated. Following this, those probesets with < 50% of their associated probes remaining were systematically removed from any transcript clusters with which they were associated. Once ≥ 50% of probesets in a transcript cluster had been removed the transcript cluster was then selected as a candidate for masking.

### Cross-species hybridisation derived experimentally

An experimental approach was employed to examine cross-hybridisation of mouse mRNA to the Human gene array in the absence of human RNA. Three independent non-engrafted mouse spleen (NES) samples were hybridised to the Human Gene 1.0 ST array, CEL files were imported into GeneSpring version GX 10.0.2 and normalised using the Robust Multi-Array (RMA-16) algorithm. The mean signal intensity of the three biological replicates was used to identify cross-hybridising transcript clusters.

## Authors' contributions

ALS wrote the manuscript, collected, analysed and summarised the data and principally managed the project. VKP extracted RNA, labelled, hybridised and scanned all microarrays, performed analysis using Genespring and R and generated the experimental masking approach. RAP maintained mouse lines, dissected tissue and prepared tissue for RNA extraction. MJF participated in the design of the statistical approach and performed statistical analysis using R. RWF designed and generated the *in silico *masking approach. ALS, AHB, URK and RBL made substantial contribution to the conception and design of the experiments. AHB, RBL and URK supported the research and provided significant intellectual contributions to the project and manuscript. All authors read and approved the manuscript.

## Supplementary Material

Additional file 1 **Pearson correlation coefficient matrix**. This file contains the Pearson correlation coefficient matrix for transcript cluster expression levels between all transcript clusters for engrafted ALL-16 xenografts and mixing of ALL-16 xenografts with mouse cells (NES). Click here for file

Additional file 2 **Transcript clusters identified as susceptible to cross-species hybridisation by experimental and *in silico *masking approaches**. This file contains the transcript cluster identity, gene title, gene symbol, chromosomal location and method used for the detection of the 1280 transcript clusters identified as susceptible to cross-species hybridisation. Click here for file
